# Pin1 Modulates the Type 1 Immune Response

**DOI:** 10.1371/journal.pone.0000226

**Published:** 2007-02-21

**Authors:** Stephane Esnault, Ruedi K. Braun, Zhong-Jian Shen, Zhuzai Xiang, Erika Heninger, Robert B. Love, Matyas Sandor, James S. Malter

**Affiliations:** 1 Department of Pathology and Laboratory Medicine, University of Wisconsin, Madison, Wisconsin, United States of America; 2 Waisman Center for Developmental Disabilities, Madison, Wisconsin, United States of America; 3 Department of Surgery, University of Wisconsin, Madison, Wisconsin, United States of America; New York University School of Medicine, United States of America

## Abstract

**Background/Abstract:**

Immune responses initiated by T cell receptor (TCR) and costimulatory molecule mediated signaling culminate in maximal cytokine mRNA production and stability. The transcriptional responses to co-stimulatory T cell signalling involve calcineurin and NF-AT, which can be antagonized by interference with the *cis-trans* peptidyl-prolyl isomerases (PPIase), cyclophilin A and FKBP. Signalling molecules downstream of CD28 which are essential for the stabilization of cytokine mRNAs are largely unknown.

**Methodology/Principal Findings:**

We now show that Pin1, a third member of the PPIase family mediates the post-transcriptional regulation of Th1 cytokines by activated T cells. Blockade of Pin1 by pharmacologic or genetic means greatly attenuated IFN-γ, IL-2 and CXCL-10 mRNA stability, accumulation and protein expression after cell activation. *In vivo*, Pin1 blockade prevented both the acute and chronic rejection of MHC mismatched, orthotopic rat lung transplants by reducing the expression of IFN-γ and CXCL-10. Combined transcriptional and post-transcriptional blockade with cyclosporine A and the Pin1 inhibitor, juglone, was synergistic.

**Conclusions/Significance:**

These data suggest Pin1 inhibitors should be explored for use as immunosuppressants and employed with available calcineurin inhibitors to reduce toxicity and enhance effectiveness.

## Introduction

During and after antigen presentation, inflammatory cytokines structure the type and amplitude of the T cell response. Especially important are the type 1 pro-inflammatory cytokines, IFN-γ, IL-2 and CXCL-10, which are produced in large amounts after infection [1] and during allograft rejection [2]. IL-2 promotes the proliferation and differentiation of T cells whereas IFN-γ activates immune cells and the antigen presentation pathway [3]. CXCL-10 (IFN-γ-inducible protein-10) is a potent chemoattractant for inflammatory cells and modulates adhesion molecule expression. The production of IFN-γ and IL-2 by T cells involves both transcriptional and post-transcriptional regulation with attenuation of either capable of significantly muting cytokine production [4], [5]. The immunosuppressives cyclosporine A (CsA) and FK506 which bind to Cyclophilin A (CyA) and FKBP12, respectively, prevent NF-AT mediated cytokine gene transcription [6]. CyA and FKBP12 are members of the peptidyl-prolyl isomerase (PPIase) family that catalyze the *cis-trans* isomerization of X-Pro peptide bonds. A third PPIase, Pin1, binds to and isomerizes only phosphorylated Ser/Thr-Pro motifs. Isomerization-induced conformational changes alter target protein function, phosphorylation and turnover [7], [8]. The initially identified Pin1 targets were cell cycle regulators particularly mitotic kinases [9]. Consistent with such a function, overexpression of Pin1 has been observed in many cancers, and its levels are predictive of cancer recurrence [10], [11]. Recently, we implicated Pin1 in the post-transcriptional control of GM-CSF mRNA by activated eosinophils and T lymphocytes [12], [13]. GM-CSF is a prototypical proinflammatory cytokine, whose mRNA is regulated by 3′-untranslated AU-rich elements (AREs). These are also found in and important for the post-transcriptional control of IL-2 and IFN-γ mRNAs [14], [15] suggesting a role for Pin1 in the type 1 immune response. In the present report, we show that Pin1 KO mice show an alternated cytokine response, after co-stimulation with anti-CD3 and anti-CD28. This reflects an inability of T cells to fully stabilize ARE mRNAs after cell activation. We explore the biology significance of these observations by testing if Pin1 blockade would alter type 1 immune responses to mismatched organ transplants. We show that mismatched lung transplants are not rejected if Pin1 is inhibited. Further, we show that Pin1 blockade is synergistic with calcineurin inhibitors such as Cyclosporin A. These data establish a new role for Pin1 in the T cell immune response and point to a novel target for immunosuppression.

## Results

**Figure 1:**
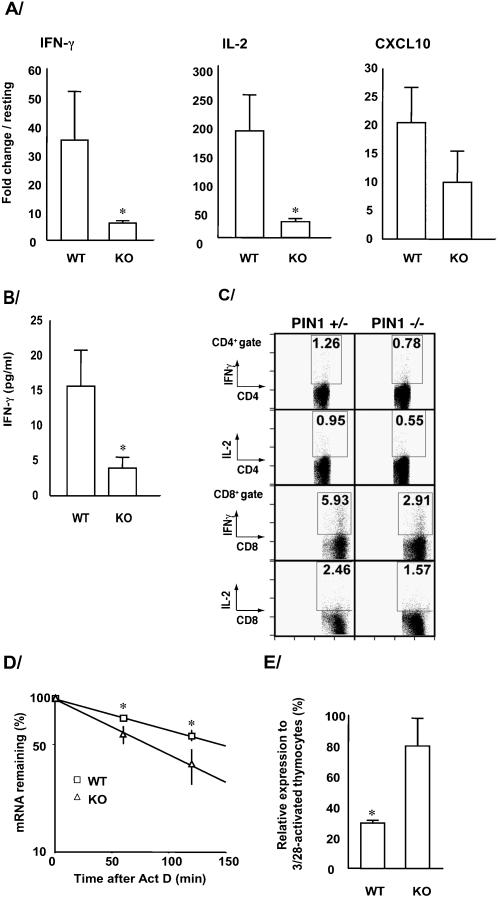
*Cytokine expression is downregulated in Pin1−/− mouse*. A/ Splenocytes from Pin1−/−mice (KO) or wild-type mice (WT) were activated for 3 hours with anti-CD3 plus anti-CD28. mRNAs for IFN-γ, IL-2 or CXCL10 were analyzed by reverse transcription, qPCR. Fold change increase in activated compared to resting cells were assessed and averages±SD from 3 mice in each groups are presented. B/ Splenocytes were activated as in (A) for 5 hours and IFN-γ amounts in the cultures were measured by ELISA. Averages±SD from 3 mice in each groups are presented. C/ Percentage of IFN-γ and IL-2 positive CD4+ and CD8+ cells in splenocytes activated for 5 hours with anti-CD3. Splenocytes were incubated for 30 min at 4°C with relevant antibodies at saturation and then washed and analyzed using a FACSCalibur instrument (Becton Dickinson, Mountain View, CA). KO mice (−/−) are compared to heterozygote mice (+/−) and a representative experiment is shown D/ Splenocytes from the same mice (n = 3) were activated with anti-CD3 plus anti-CD28 for 3 hours before treated with actinomycin D (Act D;10 µg/ml). mRNA levels were determined by qPCR at the indicated times after Act D. IFN-γ mRNA levels at T_0_ were normalized to 100%. E/ Thymocytes from Pin1−/− (KO) or wild-type (WT) mice were activated with anti-CD3 plus anti-CD28 for 3 hours with or without juglone (1 µM). mRNA levels were determined by qPCR and presented as relative (%) expression of IFN-γ in juglone-treated cells compared to activated. * denotes p<0.05.

Pin1 function on type 1 cytokine and chemokine expression was first evaluated in Pin1 knockout (KO) mice. Splenocytes from KO mice activated with anti-CD3 plus anti-CD28, which normally triggers cytokine mRNA stabilization and accumulation [4], [13], showed significantly less IFN-γ and IL-2 mRNA compared to WT (p<0.03 and p<0.008, respectively) while CXCL-10 mRNA was reduced by 50% but did not quite reach significance (**figure 1A**). Secreted IFN-γ was proportionally reduced (4-fold) in the supernatant of KO splenocyte cultures compared to WT (**figure 1B**). Bulk analysis of activated KO CD4+ or CD8+ splenocytes by flow cytometry showed reductions in IL-2 and IFN-γ positive cells (**figure 1C**) compared to splenocytes from heterozygote mice. In KO mice, no differences were noted in the numbers of splenic or thymic CD3, CD4, CD8 or regulatory T cell populations or activation marker expression after stimulation (**not shown**), eliminating developmental differences between WT and KO mice. As CD3 mediated signaling is necessary for T cell development, these data suggest TCR function is likely normal in Pin1 KO animals. Instead, these data suggested Pin1 was involved in co-stimulatory-CD3/CD28 signaling. Indeed, IFN-γ and IL-2 mRNAs were less stable in anti-CD3/anti-CD28 activated KO than WT splenocytes while the stability of CXCL-10 mRNA, which lacks AREs was unchanged (**figure 1D** **and not shown**). Therefore, Pin1 is necessary for ARE mediated cytokine mRNA stabilization after T cell co-stimulation. As Pin1 substrates also include NF-κB and NF-AT [16], which regulate cytokine mRNA transcription, the observed reductions in CXCL-10 suggest a nuclear event.

**Figure 2:**
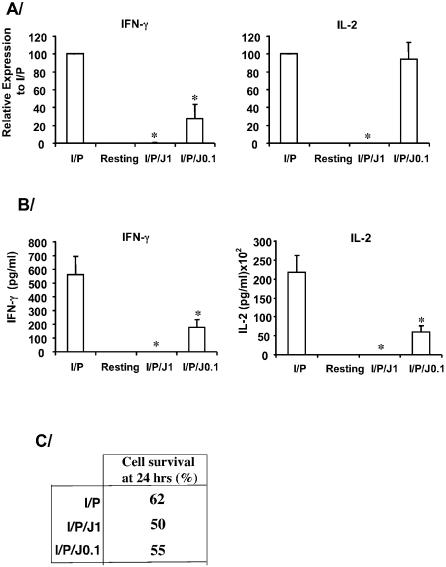
*Cytokine mRNAs are suppressed by Pin1 inhibition in activated rat splenocytes.* A/ mRNAs for IFN-γ, IL-2, and CXCL-10 were analyzed in splenocytes by reverse transcription, qPCR. Cells were cultured for 4 hours without activation (resting), or with ionophore plus PMA (I/P) or I/P plus juglone (I/P/J) at 1 or 0.1 µM. The ionomycin/PMA stimulated sample was normalized to 100 and others expressed as a % of that value. The data are averages±SEM of 3 independent experiments using splenocytes of untreated healthy animals. B/ Secreted IFN-γ and IL-2 after 24 hours from the cultures as described in (A). The data are averages±SEM of 3 independent experiments using splenocytes of untreated healthy animals. C/ Viability of rat splenocytes treated as above for 24 hr, incubated with propidium iodine and analyzed by flow cytometry.

Pin1 can be selectively blocked by juglone (5-hydroxy-1,4-naphthoquinone), which covalently binds to and irreversibly inhibits Pin-1 activity but has no effect on CyA or FKBP [17]. Prior to its use *in vivo*, WT and KO thymocytes were activated with anti-CD3/anti-CD28 while rat splenocytes were treated with ionophore/PMA (I/P) (**figures 1E** **,** **2** **and not shown).** After 4 hours of I/P, cytokine mRNAs increased by as much as 500 fold (IFN-γ. IFN-γ and IL-2 mRNA levels were reduced in the rat or mouse WT cells by 70–95% after treatment with juglone (**figures 1E** **,** **2A, and 2B**). However, juglone had no significant effect on the abundance of IL-2, IFN-γ or the housekeeping RNAs in Pin1 KO cells (**figure 1E** **and not shown**), demonstrating the specificity of this drug. At the concentrations used here neither CyA nor FKBP12 peptidyl-prolyl isomerase activities are affected [17]. Juglone (1 µM) completely blocked the accumulation of I/P-induced IFN-γ and IL-2 mRNAs by rat splenocytes without altering actin (**figure 2A**). At lower juglone concentrations (0.1 µM), only the accumulation of IFN-γ mRNA was significantly inhibited (**figure 2A**). These data suggest a variable sensitivity of cytokine mRNAs to Pin1 inhibition. The accumulation of IL-2 and IFN-γ after juglone were dramatically reduced in the culture supernatants (**figure 2B**). The failure to produce cytokines was not a function of cell death as equivalent levels of viability were observed in I/P versus I/P/juglone treated splenocytes (**figure 2C**).

**Figure 3:**
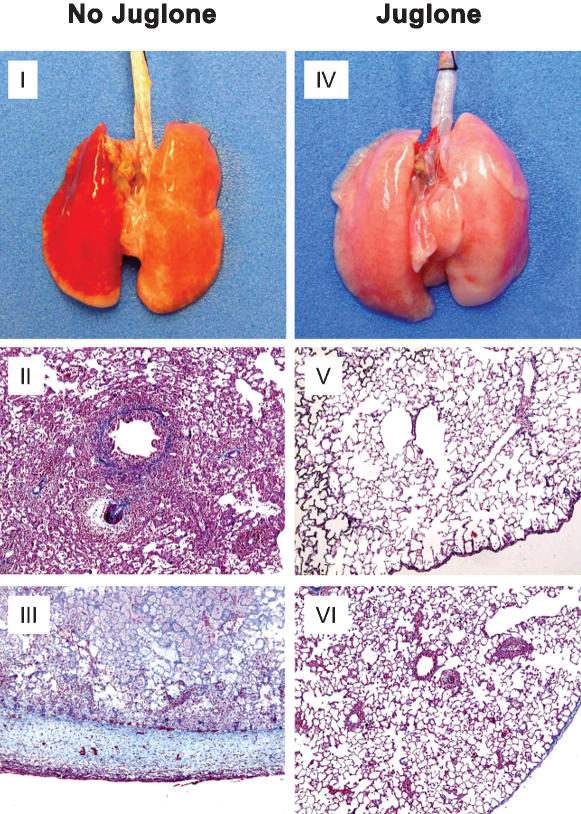
*Pin1 is required for acute and chronic rejection.* I and IV: Gross appearance of lungs from control, untreated transplants (I) and juglone treated (1 mg/kg) (IV). Lungs are oriented so that transplant is on the left. II and V: Hematoxylin and eosin stained sections from control untreated (II) or juglone treated (V) 1 week after transplant. III and VI: Trichrome stained sections of control, untreated (III) and juglone treated (VI) two weeks after transplant. These are representative sections from 8 control and 8 juglone treated transplant recipients in each set.

**Figure 4:**
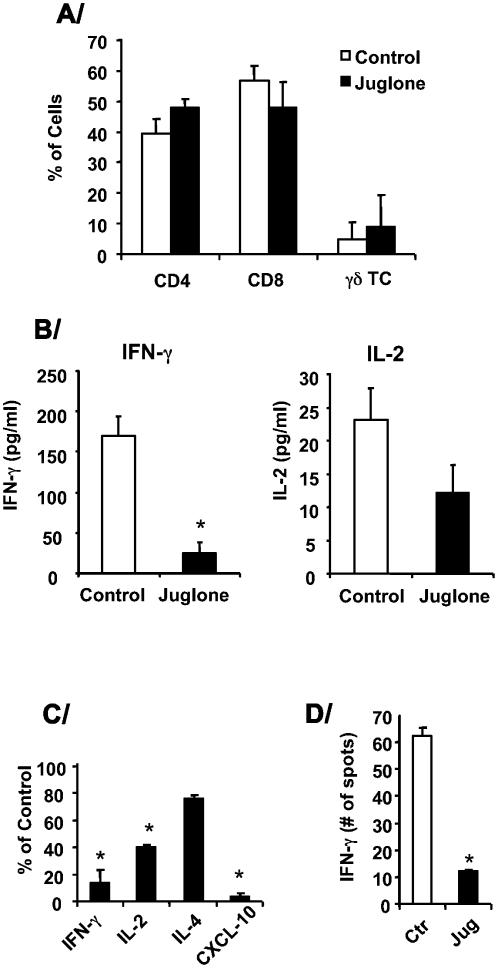
*IFN-γ and IL-2 are Pin1 dependent.* A/ Relative percentage of CD4, CD8 and γδ T lymphocytes in BAL cells from the transplanted lung. BAL cells were prepared from control and juglone-treated rats as described above and the percentage of CD4+, CD8+ and γδ T lymphocytes were determined by flow cytometry with specific antibodies. The graph represents an average +/− SD of 4 experiments. B/ IFN-γ and IL-2 concentrations in concentrated BAL fluid were determined by ELISA. * denotes p<0.01. C/ Mediastinal lymph nodes from control or juglone treated animals were used for reverse transcription, qPCR for the cytokines shown along the x-axis. The values in untreated controls were normalized to 100% and data shown represent % change in juglone treated samples. In (B) and (C), the data shown are an average±SEM of 4 rats in each group 7 days after transplantation. * denotes p<0.05 from control samples. D/ Splenocytes were isolated and cultured as described in Material and Methods. IFN-γ positive splenocytes were quantitated by Elispot (n = 3). * denotes p<0.05.

In order to characterize Pin1 function during an *in vivo* type I immune response, we used the widely employed F344 to WKY rat, MHC Class I mismatched, orthotopic, single lung transplantation model [18], [19]. The donor organ is attached via cuffs to the recipient's bronchial and vascular systems permitting normal function. Nonimmunosuppressed recipients experience profound acute rejection within several days largely mediated by IFN-γ and CXCL-10 upregulation [20]–[24]. Over several weeks, chronic rejection occurs with alveolar, pleural, and peribronchial collagen deposition, loss of viable pneumocytes and eventual organ loss. Recipients were given a daily, single intraperitoneal (IP) injection of 1 mg/kg juglone dissolved in ethanol and diluted in 5 ml saline, while controls received diluents only. This doses of juglone had no effect on red cell mass, white cell counts, serum chemistries, liver function tests, or renal function in control, untransplanted rats (**not shown)**. Therefore juglone administration appeared to be safe and nontoxic. Treatment was started the day of the transplant. At day 7 or 14, animals were sacrificed and the lungs evaluated grossly and by histopathology. In untreated animals, the transplant was visibly shrunken and the pleural surfaces hemorrhagic (**figure 3**). Palpation revealed a firm and unyielding consistency. The juglone treated animals showed no gross signs of rejection (**figure 3**) and was indistinguishable from the contralateral control. Microscopically, the untreated transplanted lung showed severe rejection with acute inflammatory cell infiltration predominantly composed of neutrophils, lymphocytes and macrophages. Alveolar architecture was totally effaced and the small airways packed with inflammatory cells (**figure 3**). These changes were completely absent in juglone treated animals, which showed normal alveolar architecture, pleural thickness, and airway patency (**figure 3**). Occasional round macrophages were present in some alveoli. No significant difference was found in the relative proportion of CD4, CD8 or γδ T cells in blood and BAL fluid from the native right lung or the transplanted left lung, 7 days after transplantation in juglone treated or untreated animals **(not shown and** **figure 4A** **)**. This is in contrast to collagen V tolerized animals that show predominantly CD4^+^ T cells [25]. These data demonstrate that Pin1 blockade can dramatically attenuate acute transplant rejection.

**Figure 5:**
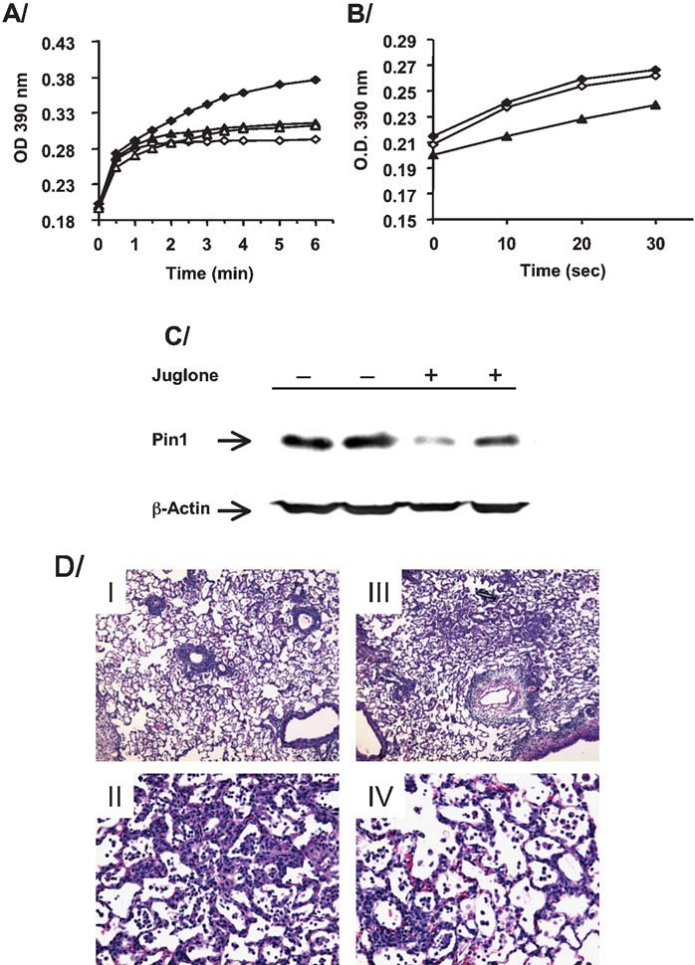
*Expression of exogenous IFN-γ and CXCL-10 in juglone-treated rats overcomes Pin1 inhibition and results in rejection*. A/ BAL fluid cells from transplanted and untreated controls (⧫), or juglone treated (▴) were quantitated, lysed and used for Pin1 isomerase assay. Juglone (1 µM) was also added *in vitro* to equal amounts of the control (⋄) and juglone treated (▵) samples and isomerase assay repeated. B/ Mediastinal lymph nodes from 2 untreated controls (⧫,⋄) or one juglone treated animal (▴) were lysed and equal amounts of protein used for Pin1 isomerase assay. C/ Immunoblot with anti-Pin1 or anti-actin of mediastinal lymph nodes from 2 controls, untreated (−juglone) or 2 juglone treated animals (+juglone). D/ Donor lungs were insufflated with 100 µg each of CXCL10 and IFN-γexpression vectors immediately prior to religation in recipient. Animals were then untreated (I, II) or treated with juglone at 1mg/kg for 1 week (III, IV) and histopathologic analysis after H & E staining. Representative sections from multiple animals shown.

One week after transplantation the levels of IFN-γ, and IL-2 in the bronchoalveolar fluid (BAL) were reduced (**figure 4B**). These data suggest that Pin1 blockade prevented cytokine production by T cells, which were the majority population within the BAL fluid (**not shown**). Since draining lymph nodes are a predominant site for immunological reactivity we analyzed mediastinal lymph node cells for the expression of cytokine mRNAs by qPCR. mRNAs coding for IFN-γ, IL-2, and CXCL-10 were significantly lower in juglone treated animals than controls whereas IL-4 was not significantly affected (**figure 4C**). IFN-γ ELISPOTS of whole spleen showed large reductions in the juglone treated animals from untreated controls (**figure 4D**). At day 7, Pin1 PPIase activity and amount were attenuated in BAL cells and draining lymph nodes in juglone treated rats (**figure 5A–C**). Once inactivated by juglone, Pin1 is rapidly degraded by the proteasome [12]. Therefore, Pin1 inhibition prevented IFN-γ, and IL-2 expression by T cells throughout the immune system and acute graft rejection after lung transplantation. The profound suppression of CXCL-10 presumably reflects both reductions in IFN-γ, which normally induces CXCL-10 as well as direct effects of Pin1 on CXCL-10 expression.

The expression of these cytokines has been highly correlated with rejection in both animal models and humans [20], [21]. In order to demonstrate a causal role, IFN-γ and CXCL-10 expression vectors were combined and insufflated into donor lungs immediately before religature in the recipient. To avoid potential Pin1 mediated, post-transcriptional regulation, only the coding region without the 3′ UTR was inserted downstream of a constitutively active CMV promoter. By 1 week after transplant, the lung grafts underwent significant rejection in untreated controls, which was prevented by juglone (**figure 3**). The forced expression of IFN-γ and CXCL-10 in juglone treated rats resulted in severe cellular infiltration irrespective of Pin1 blockade (**figure 5D**). Transgenic cytokines were detectable in the BAL at comparable levels to that seen in untreated recipients (**not shown**). These results support a central role of IFN-γ and CXCL-10 in the process of acute rejection and suggest that cytokine suppression after Pin1 inhibition is likely responsible for graft sparing.

**Figure 6:**
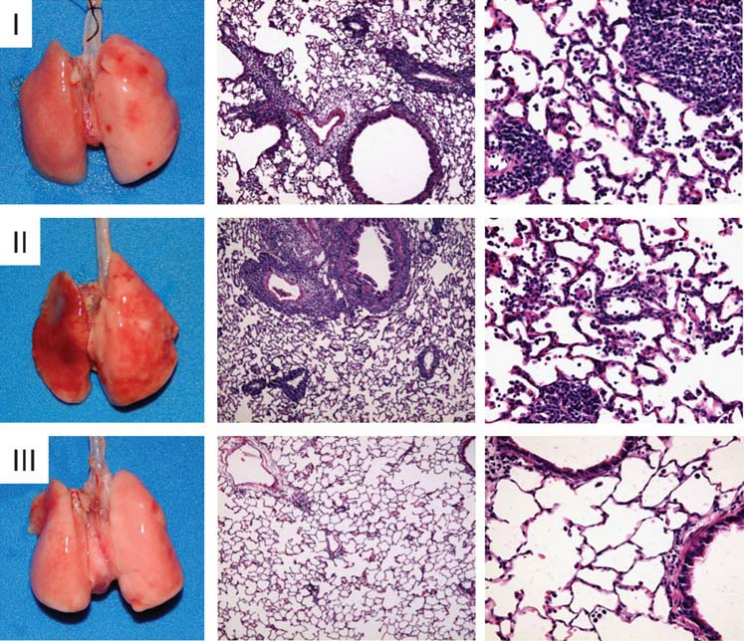
*Pin1 and calcineurin inhibition are synergistic.* Transplants were performed as for figure 3 and animals harvested 7 days later. (I) Treated with CsA at 1 mg/kg/d for 3 days. (II) Treated with juglone at 0.1 mg/kg/d for 7 days. (III) Treated with CsA at 1 mg/kg/d for 3 days plus juglone at 0.1 mg/kg/d for 7 days. For all conditions, gross apearance of the transplant at harvest shown along the left, 5× and 20× representative sections stained with H & E shown in the middle and right panels.

Immunosuppressive drugs, particularly CsA greatly improves graft survival but have renal damage as a common side effect [26]. To avoid toxicity and enhance graft acceptance, immunosuppressants such as corticosteroids, calcineurin inhibitors, antimetabolites, and rapamycin are given in combination [26]. Cyclosporine A and tacrolimus indirectly inhibit nuclear factor of T cells (NFAT) preventing the transcriptional upregulation of cytokine genes by activated T cells [26]. In contrast, Pin1 suppresses expression through the regulation of cytokine mRNA stability. Given this distinct mode of action, we asked if combined CsA and juglone therapy would be additive or synergistic. CsA was injected intraperitoneally for 3 days at a dose of 1 mg/kg together with 0.1 mg/kg of juglone, which was then continued alone for 4 more days. Cyclosporine is usually used at 25 mg/kg/day for 3 days which induces long lived lung transplant acceptance in rats while moderate to severe rejection was observed at doses of 5 mg/kg/d [27]. Transplanted animals treated with CsA alone (1 mg/kg/d) showed modest graft discoloration (**Figure 6**) but microscopically severe cellular infiltration diffusely throughout the parenchyma with foci in peribronchial and perivascular areas (**Figure 6**). Similarly, suboptimal inhibition of Pin1 leads to severe rejection pathology with substantial cellular infiltration and hemorrhage (**Figure 6**). In contrast, combined suboptimal treatment with CsA and juglone provided excellent graft protection without identifiable cellular infiltrates (**Figure 6**). These data show that combined inhibition of Pin1 and calcineurin are additive or synergistic and suggest that CsA dosage could be signifcantly reduced if Pin1 inhibition is added to the therapeutic regiment.

## Discussion

We recently reported that Pin1 plays a role in the post-transcriptional control of GM-CSF mRNA expression by activated eosinophils and lymphocytes. Mechanistically, Pin1 interacts with and controls the ARE mRNA-binding activity of AUF1. When associated with AUF1, ARE mRNAs such as GM-CSF are rapidly catabolized by the exosome preventing translation. Cell activation rapidly increased Pin1 activity leading to AUF1 isomerization and loss of mRNA binding. Under these conditions, GM-CSF mRNA was stabilized and translated [12], [13]. These data suggested that other cytokine mRNAs, particularly those with AREs in their 3′ untranslated region might be similarly regulated. Here we show *in vitro*, using either Pin1 KO or juglone-treated splenocytes from WT rats that Pin1 activity is required to stabilize, accumulate and translate mRNAs coding for several type-1 cytokines including IFN-γ and IL-2. These effects on IFN-γ and IL-2 were observed *in vivo* after juglone treatment of rats receiving MHC Class I mismatched, orthotopic, single lung transplants. CXCL-10 *in vitro* and IL-4 *in vivo* mRNA levels were far less affected by Pin1 blockade, suggesting Pin1 regulates only the most labile AU-rich mRNAs. These results are in agreement with our previous study showing IL-4 expression is insensitive to Pin1 inhibition [13], and reinforces the conclusion that Pin1 affects mRNA levels specifically through ARE-binding proteins [12], [13]. *In vivo*, the profound suppression of CXCL-10 observed presumably reflects both reductions in IFN-γ, which normally induces CXCL-10 as well as direct effects of Pin1 on the production of CXCL-10, which might include nuclear events [16]. Finally, incomplete Pin1 blockade using low dose juglone [13] failed to decrease IL-2 mRNA levels but significantly reduced protein secretion. Therefore, in addition to stabilization and transcription, Pin1 may affect the of ARE mRNAs as well.

Pin1 differs from CyA and FKBP12 in uniquely recognizing phosphorylated Ser/Thr-Pro motifs via a group IV WW domain and its PPIase is structurally distinct [28]. Unlike CyA and FKBP12, Pin1 is sensitive to juglone, which irreversibly binds to cysteines within the isomerase domain [17]. Enzymatic assays herein demonstrated that Pin1 activity in cell lysates most proximal to allostimulation (BAL and mediastinal nodes) was completely suppressed. *In vitro*, 5.7 µM juglone eliminated essentially 100% of Pin1's and highly related yeast and *E*. coli parvulin isomerases activity [17]. Importantly, these juglone concentrations had no effect on cyclophilin A nor FKBP12 [17]. Assuming complete distribution throughout all body water, the 1 mg/kg dose of juglone used here should generate a maximal concentration of ∼10 µM. Therefore, it is very unlikely that cyclophilin A nor FKBP12 were affected by juglone treatment and underlie the graft acceptance seen here. While juglone has been shown to affect other enzymes including RNA polymerase, pyruvate decarboxylase and glutathione S-transferase [29], none of these has been implicated in the selective expression of the subset of cytokine mRNAs downregulated in these experiments. Were these enzymes or polymerases inhibited, global rather than selective downregulation of gene expression would be expected. Therefore, we conclude that the immunosuppressive effects of juglone *in vivo* reflect Pin1 blockade. In addition, the specificity of juglone on IFN-γ and IL-2 expression through Pin1 has been confirmed using T cells from KO mice. Indeed, juglone did not affect IFN-γ and IL-2 expression in activated T cells from these animals. Importantly, these doses of juglone were nontoxic. Neither renal, hematology nor liver function tests showed abnormalities after treatment for 2 weeks. While it is unlikely juglone would be employed clinically, these data are encouraging for the development of additional Pin1 inhibitors.

Irrespective of molecular mechanism, suppression of Pin1 clearly prevented acute and chronic graft rejection. Most prominently absent were inflammatory cells and compensatory alveolar, pleural and bronchial fibrosis. Based on our data, we favor three explanations for graft survival after Pin1 inhibition. First, the production or expression of critical, proinflammatory chemokines (CXCL-10) and cytokines (IFN-γ and IL-2) containing 3′ untranslated region AREs were markedly attenuated. CXCL-10 levels have been well correlated with the rejection by human recipients of renal, cardiac and lung allografts [20], [21], [30]. Cardiac allografts were not rejected if derived from CXCL-10 deficient donor mice [22] nor if wild type organs were transplanted into CXCL-10 receptor knockout recipients [31]. Similarly, IFN-γ and CXCL-10 have been highly associated with rejection in human cardiac and lung allograft recipients [32]–[34] and the appearance of IFN-γ positive splenocytes predicted the onset of renal transplant rejection [35]. Therefore, IFN-γ and CXCL-10 appear particularly important for T cell chemotaxis into and propensity to initiate an anti-graft response. This was directly tested here by constitutive overexpression of IFN-γ and CXCL-10 within the graft and demonstration that Pin1 blockade was unable to prevent immune cell infiltration and rejection. Finally, the lack of chronic rejection and collagen deposition observed here may reflect a role of Pin1 in TGF-β1 production or signalling to fibroblasts. TGF-β1 induced collagen III mRNA was significantly reduced by escalating doses of juglone (Shen *et al*., manuscript submitted). The mechanism underlying this observation is unknown but presumably involves Smad signalling. These data suggest Pin1 blockade could be effective in reducing the fibrosis characteristic of BOS, which is seen despite immunosuppression with CsA, steroids and other agents.

The identification of Pin1 as a potential target for immunosuppression could have considerable importance. Most currently employed drugs have been available for 15 years or more and despite combined therapy, continue to have significant side-effects. Based on the data here and previously [12], [13], Pin1's regulation of immune responses involves post-transcriptional pathways largely unaffected by CsA or FK506. Therefore, we tested if CsA plus juglone showed additive effects. Indeed, combined therapy with suboptimal doses of CsA and juglone showed dramatic inhibition of graft rejection, demonstrating that these agents could be used together with effectiveness and possibly lower toxicity.

The data presented here show that Pin1 influences the type I immune response by modulating the production of proinflammatory, T cell cytokines including IFN-γ and IL-2. Based on prior work, Pin1 likely functions through direct interactions with and regulation of key AUUUA specific, mRNA binding proteins such as AUF1, HnRNP C and HuR [12], [13]. Interference with Pin1 through pharmacologic or genetic means reduced IFN-γ and IL-2 production and substantially attenuated graft rejection. Our data predict that drug combinations directed at the production (CsA, FK506, steroids) and decay of cytokine mRNAs would more effectively suppress alloimmunity and do so with reduced toxicity. More generally, the broad range of type 1 immune responses such as infectious diseases (intra and extracellular pathogens) or autoimmunity (diabetes, multiple sclerosis and lupus) that involve or require IFN-γ suggest a role for Pin1 in these processes as well.

## Materials and Methods

### Reagents

Juglone (5-hydroxy-1,4-naphthoquinone), ethanol, ionomycin and phorbol myristate acetate (PMA) were from Sigma (Sigma-Aldrich Chemical Company, St. Louis, MO), isoflurane was from Phoenix Pharmaceutical Inc. (St. Joseph, MO). Anti-CD3-coated plates, control plates and the anti-CD28 antibody were provided by BD Biosciences, Bedford, MA. Cyclosporine A was purchased from Gallipot, Inc. (St. Paul, MN) through the hospital pharmacy.

### Pin1 −/− mice

Pin1 null mice were originally generated by Fujimori, and the Pin1 deletion was transferred into an isogenic C57BL/6J background by Jackson Laboratory. Dr. Anthony Means of Duke University Medical Center generously provided Pin1^+/−^ mice to our laboratory. Mice were housed 2–4 per microisolator cage on a 6am–6pm light cycle with ad libitum food (Purina 5015 mouse diet) and water. The cages contained seeds and a neslet as the only sources of environmental enrichment. All husbandry and euthanasia procedures were performed in accordance with NIH and an approved University of Wisconsin Madison animal care protocol through the Research Animal Resources Center. As homozygous Pin1 mutant mice are infertile, heterozygote mice were mated to generate WT, heterozygote and knock-out progeny. Genotypes were determined by PCR analysis of DNA extracted from tail biopsies taken at the time of weaning (postnatal day 18). The forward 5′-ATCATCCTGCGCACAGAATG-3′ and reverse 5′-TCAATTCCTCCAGAAGGAGC-3′ primers for the WT pin1 allele and the forward 5′-CTTGGGTGGAGAGGCTATTC-3′ and reverse 5′-AGGTGAGATGACAGGAGATC-3′ primers for the disrupted allele produced 195 and 280 nt PCR products, respectively. Thymus and spleen were dissected from adult mice (12 weeks old). Splenocyte and thymocyte suspensions were prepared using cell strainers from BD Bioscience (San Jose, CA). Red blood cells were lysed with ammonium chloride.

### Transplantation

All experiments were carried out under an approved animal protocol with F344 and WKY rats weighing 300–350 g (Harlan, Indianapolis, IN). The animals were housed with 2–3 animals per cage that were maintained in environmentally controlled rooms at 22°C and 50–55% humidity, with a 12/12h light/dark cycle. All rats were fed a standard laboratory diet (NIH-07 Rat and Mouse Chow) and were provided water ad libitum. The animal resource services facilities are accredited by the American Association for the Accreditation of Laboratory Animal Care. Animals were euthanized with an overdose of inhaled isoflurane. The orthotopic transplantation of left lung was performed as previously reported using the cuff technique [19]. Briefly: rats were anaesthetized by inhalation of a mixture of isoflurane and oxygen, intubated and ventilated with a mixture of isoflurane and oxygen to maintain anesthesia. The donor rat was placed in a supine position and the heart and lungs were removed *en bloc*. The left lung was resected and the pulmonary vein, bronchus, and pulmonary artery were passed through teflon cuffs and the proximal ends were everted over the cuffs. On the recipient rat a left thoracotomy was performed. The donor pulmonary vein, bronchus, and pulmonary artery were inserted into the corresponding recipient hilar structures, and fixed with separate circumferential ligatures of silk. The chest wall was closed, and the isoflurane was stopped.

### Juglone and cyclosporine preparation and injection

Juglone was dissolved in 100% ethanol at 14 mM (2.44 mg/ml). For injection between 100 and 150 µl of this solution (depending on the animal weight) was diluted in 5 ml saline and injected i.p. within 30 minutes. For suboptimal dosing between 10 and 25 µl of this solution was injected. Control animals received weight appropriate volume (100–150 µl; 10–25 µl resp.) of 100% ethanol in 5 ml saline. Cyclosporine A was dissolved in 100% ethanol at 25 mg/ml. For injection 10 to 25 µl of this solution (depending on the animal weight) was diluted in 5 ml saline and injected prior to the transplantation and the two following days.

### Preparation of lung, lymph nodes and blood

After sacrifice blood was drawn from the abdominal vein into a heparinized tube. BAL was performed separately in the left transplanted lung and the right native lung. Lungs were then remove *en bloc* and fixed by bronchial infusion of a 1% paraformaldehyde solution at 30 cm H_2_O pressure overnight. Mediastinal lymph nodes and spleen were removed and cell suspensions were prepared using cell strainers from BD Bioscience (San Jose, CA). Red blood cells were lysed with ammonium chloride.

### Pin1 activity assay

Activity was measured as described previously [12], [13]. White blood cells from lymph node, spleen, and bronchoalveolar lavage fluid were lysed by repeated freeze-thaw cycles in a buffer containing 50 mM HEPES and 100 mM NaCl, pH 7.0. Total protein (10 µg in 10 µl) was mixed with 70 µl of HEPES-NaCl buffer supplemented with 2 mM dithiothreitol and 0.04 mg/ml BSA. Then, 5 µl of α-chymotrypsin (60 µg/µl in 0.001 N HCl) was added and mixed throughly. Finally, 5 µl of the tetrapeptide substrate Suc-Ala-Glu-Pro-Phe-pNa (Peptides International, Louisville, Kentucky) dissolved in dimethylsulfoxide and preincubated at a concentration of 100 µg/ml in 480 mM LiCl and trifluoroethanol, was added. Absortion at 390 nm was measured over 30 min with a Beckman Coulter DU 800 spectrophotometer.

### Cytokine mRNA analysis

For the measurement of mRNA steady state levels in immune cells, 2×10^5^ to 1×10^6^ cells were lysed in TRI reagent (Molecular Research Center, Cincinnati, OH) and processed as already described [13]. Total RNA was extracted as described by the manufacturer. The synthesis of first strain cDNA was performed using the manufacturer's protocol (Qiagen Inc. Valencia, CA). Briefly, total RNA was resuspended in a final volume of 20 µl containing 4 units of Omniscript, 2 µl of 10× RT buffer, 2 µl of 5 mM dNTP, 20 units of RNase inhibitor and 1 µl of random hexamer primers (27 OD/µl) (Invitrogen, Carlsbad, CA) and incubated at 37°C for 60 min. 1/10 to 1/20 of the RT products were used for the real time PCR (qPCR). The primers were selected using the Primer Express software and the SYBR green master mix from Applied Biosystems was used as recommended by the manufacturer. For each set of primers, the efficiency of the PCR was determined after serial dilutions of the cDNA. The PCR efficiencies were > 90% and allowed the ΔΔCT calculations relative to β-actin (Rat) or 18S (mouse) in order to calculate the fold change (2^−ΔΔCT^) in mRNA steady state levels between juglone-treated or untreated groups.

### ELISPOT and ELISA

IFN-γ measurements in the mouse splenocyte cultures were determined using an EKISA kit (Biosource, Camarillo, CA). IFN-γ secretion by splenocytes from transplanted rats was evaluated by ELISPOT (R&D Systems, Minneapolis, MN) as described by the manufacturer. Triplicates of serial dilutions of cells were cultured with PMA (10 ng/ml) plus iononycin (1 µM) for 48 hours. The concentration of IFN-γ and IL-2 in the BAL fluid was determined using an ELISA kit (R&D Systems, Minneapolis, MN). Lavage fluid was concentrated 10 fold prior to analysis. Lavage recovery was similar in all animals and the data shown are those from the concentrated samples.

### IFN-γ and CXCL-10 expression vectors

pcDNA1 (InVitrogen) was opened at Xba 1 and Xho 1 sites and PCR products coding for the coding regions of IFN-γ and CXCL-10 inserted via sticky end ligation using standard methods. Putative clones were sequenced to ensure full length insert was present in the appropriate orientation. After amplification, plasmids were precipitated, washed and resuspended in sterile TE at 1 mg/ml and 100 µl used for insufflation.

### Splenocyte survival

The survival of resting, ionomycin plus PMA (I/P) without or with juglone at 1 (I/P/J1) or 0.1 µM (I/P/J0.1)-treated splenocytes were determined by propidium iodide staining and flow cytometry at 24 hours after the beginning of the culture.

### Statistics

1-sample t-tests were used for statistical analysis of the data.
